# Donor Characteristics and Regional Differences in the Utilization of HCV-Positive Donors in Liver Transplantation

**DOI:** 10.1001/jamanetworkopen.2020.27551

**Published:** 2020-12-04

**Authors:** Ben L. Da, Ghideon Ezaz, Tatyana Kushner, James Crismale, Gaurav Kakked, Ahmet Gurakar, Douglas Dieterich, Thomas D. Schiano, Behnam Saberi

**Affiliations:** 1 Division of Liver Diseases, Icahn School of Medicine at Mount Sinai, New York, New York; 2 Sandra Atlas Bass Center for Liver Diseases & Transplantation, Division of Hepatology, Department of Internal Medicine, Donald and Barbara Zucker School of Medicine for Hofstra/Northwell Health, Manhasset, New York; 3 Division of Gastroenterology and Hepatology, Johns Hopkins University School of Medicine, Baltimore, Maryland; 4 Division of Gastroenterology and Hepatology, Beth Israel Deaconess Medical Center, Harvard Medical School, Boston, Massachusetts

## Abstract

**Importance:**

Increased utilization of hepatitis C virus (HCV)–positive liver allografts for liver transplant (LT) has been endorsed as one of several ways to combat national organ shortages. However, HCV-positive donors remain poorly characterized, and Organ Procurement and Transplantation Network regional differences in the utilization of HCV-positive liver allografts are unclear.

**Objective:**

To characterize HCV-positive donors and the allografts that come from them.

**Design, Setting, and Participants:**

In this cross-sectional study, the Scientific Registry of Transplant Recipients database was queried for all donors who underwent HCV testing from June 2015 to December 2018. Clinical and allograft characteristics were evaluated, and utilization across the United States was studied. Patients with positive or negative results for HCV antibody (Ab) and HCV nucleic acid amplification testing (NAT) were included in this study. Donors utilized for living donor transplant and pediatric (age <18 years) recipients were excluded.

**Main Outcomes and Measures:**

The primary comparison was between donors who were HCV Ab positive and those who were HCV Ab negative. Regional variations in the utilization of HCV-positive and HCV-negative donors were analyzed.

**Results:**

Of 24 500 donors utilized for LT, 1887 (7.7%) were HCV Ab positive; 64.4% of HCV Ab–positive donors were HCV NAT positive. HCV Ab–positive donors were younger (median [interquartile range] age, 35 [29-46] years vs 40 [27-54] years) and had fewer comorbidities, such as diabetes (8.3% vs 12.0%) and hypertension (25.9% vs 35.2%), compared with HCV Ab–negative donors. These findings were even more pronounced in HCV Ab–positive /NAT–positive compared with HCV Ab–positive/NAT–negative donors. Organ Procurement and Transplantation Network regions 2, 3, 10, and 11 had the highest absolute utilization of HCV Ab–positive donors, accounting for 64.4% of all HCV Ab–positive donors used in the United States. Region 1 had the highest relative utilization of HCV Ab–positive donors (18.7%). The use of HCV Ab–positive donors in some regions was associated with the rate of drug overdose, but this was not always the case. Similar utilization results were found with HCV NAT–positive donors.

**Conclusions and Relevance:**

In this cross-sectional study, HCV-positive donors were younger and healthier than utilized HCV-negative donors. Significant differences exist in the utilization of HCV-positive donors across the 11 Organ Procurement and Transplantation Network regions, which is not entirely explained by organ demand or by higher availability of HCV-positive livers as per the distribution of the opioid epidemic. Initiatives to increase the use of HCV-positive donors, particularly in regions of high organ demand, should be implemented.

## Key Points

**Question:**

What are the clinical characteristics of donors who are positive for hepatitis C virus (HCV), and how are these donors utilized across the United States?

**Findings:**

This cross-sectional study found that HCV-positive donors were healthier and donated superior liver allografts compared with HCV-negative donors. There were substantial variations in the utilization of HCV-positive donors across the United States that were not entirely explained by the geography of the opioid epidemic.

**Meaning:**

These findings suggest that policies to drive the increased utilization of HCV-positive donors should be encouraged and implemented.

## Teaser

This cross-sectional study examines clinical characteristics of liver donors who are positive for hepatitis C virus (HCV) and differences in use of HCV-positive allografts in regions of the United States.

## Introduction

Despite an increase in the number of liver transplants (LTs) performed in the United States, the rate at which patients are placed on the waitlist continues to surpass the increased number of transplants, creating a significant mismatch between organ supply and demand.^1^ The use of hepatitis C virus (HCV)–positive organs has been widely adopted as one of the solutions to the major organ shortage by increasing the donor pool.^2^ Although HCV infection is estimated to affect 1% to 2% of the general US population, this risk is higher (3%-18%) among organ donors with increased risk as defined by the Public Health Service.^3^ In light of the ongoing opioid epidemic and the associated rise in drug overdose deaths, the number of Public Health Service “increased risk” HCV-positive organs available has been increasing rapidly.^4,5^

Even before the use of direct-acting antiviral (DAA) therapy for hepatitis C, large comparative outcome studies of patients with HCV cirrhosis showed comparable outcomes when receiving liver allografts from HCV-positive vs HCV-negative donors.^6,7^ With the widespread use of DAAs, there has been a rapid rise in the utilization of HCV-positive liver allografts.^8^ Based on Organ Procurement and Transplantation Network (OPTN) data, between 2016 and 2018, 7.4% of all deceased LT donors were HCV positive, representing a rise of 45% compared with the previous 3-year period (2013-2015).^9^ One of the reasons for this rise is the increasing utilization of HCV-positive donors for HCV-negative recipients, with studies reporting favorable results, including improved survival for recipients with a Model for End-Stage Liver Disease (MELD) score of 20 or greater due to less waitlist mortality.^10,11,12^ However, there have been limited studies on the characteristics of HCV-positive donors and allografts.

Despite advances in treatment, a large number of HCV-positive liver allografts, in the several hundreds per year, are still not being utilized.^13^ As such, there is reason to believe that individual centers and regions are utilizing more of the available HCV-positive organs than others. For instance, Verna et al^14^ recently reported that half of solid organ (kidney, liver, heart, and lung) transplants from HCV-positive donors to HCV-negative recipients occurred at 8 centers.

In this study, we aimed to characterize HCV-positive compared with HCV-negative donors of transplanted liver allografts using the Scientific Registry of Transplant Recipients (SRTR) database. In addition, we aimed to identify city and regional differences in the utilization of HCV-positive liver allografts in the United States with a focus solely on the utilization of donors. In doing so, we intended to identify the challenges contributing to the underutilization of HCV-positive liver allografts. We believe these data are essential to drive the increased use of HCV-positive donors moving forward.

## Methods

### Study Design and Patient Population

We studied all deceased LT donors that underwent hepatitis C antibody (HCV Ab) testing and HCV nucleic acid amplification testing (HCV NAT) from June 1, 2015, to December 1, 2018. The start date of our study corresponds to 3 months after HCV NAT test results began to be collected for all patients in the SRTR data set, allowing for comparison between HCV Ab–positive and HCV NAT–positive donors. Donors were excluded if they had unknown or undetermined HCV Ab or HCV NAT results, were utilized in living donor living transplant (LDLT), or had a pediatric recipient (<18 years old).

Our primary comparison was between donors who were HCV Ab positive and those who were HCV Ab negative. Although there was a recent consensus in defining HCV-positive donors as those who are NAT positive,^13^ we chose a serologic definition as there is typically a delay in testing time for NAT (30-40 minutes for serology vs up to 12-24 hours for NAT), which may affect donor procurement decisions.^15^ We also conducted secondary analyses comparing those who were HCV NAT positive vs HCV NAT negative, as well as a comparison of HCV Ab–positive/NAT-positive vs HCV Ab–positive /NAT-negative donors. Regional variations in the utilization of HCV-positive and HCV-negative organs were evaluated. The eMethods in the Supplement include details on data collection.

### Data Source

This study used data from the SRTR. The SRTR data system includes data on all donor, waitlisted candidates, and transplant recipients in the United States, submitted by the members of the OPTN. The Health Resources and Services Administration of the US Department of Health and Human Services provides oversight to the activities of the OPTN and SRTR contractors.

The data reported here have been supplied by the Minneapolis Medical Research Foundation as the contractor for the SRTR. The interpretation and reporting of these data are the responsibility of the authors and in no way should be seen as an official policy of or interpretation by the SRTR or the US government. As the SRTR is a publicly available deidentified patient-level data set, institutional review board approval was not required according to the policies of SRTR or Mount Sinai.

### Statistical Analysis

Categorical variables are reported by percentages, and continuous variables are reported as medians with interquartile ranges (IQR). Differences between categorical variables were analyzed with the χ^2^ test. Differences between quantitative variables were analyzed with a Kruskal-Wallis test. Statistical analyses were performed using the STATA software package (version 15.1; StataCorp). The threshold for statistical significance was *P* < .05 using 2-tailed tests.

## Results

### The Trend of HCV Positive Donors

**Figure 1.**
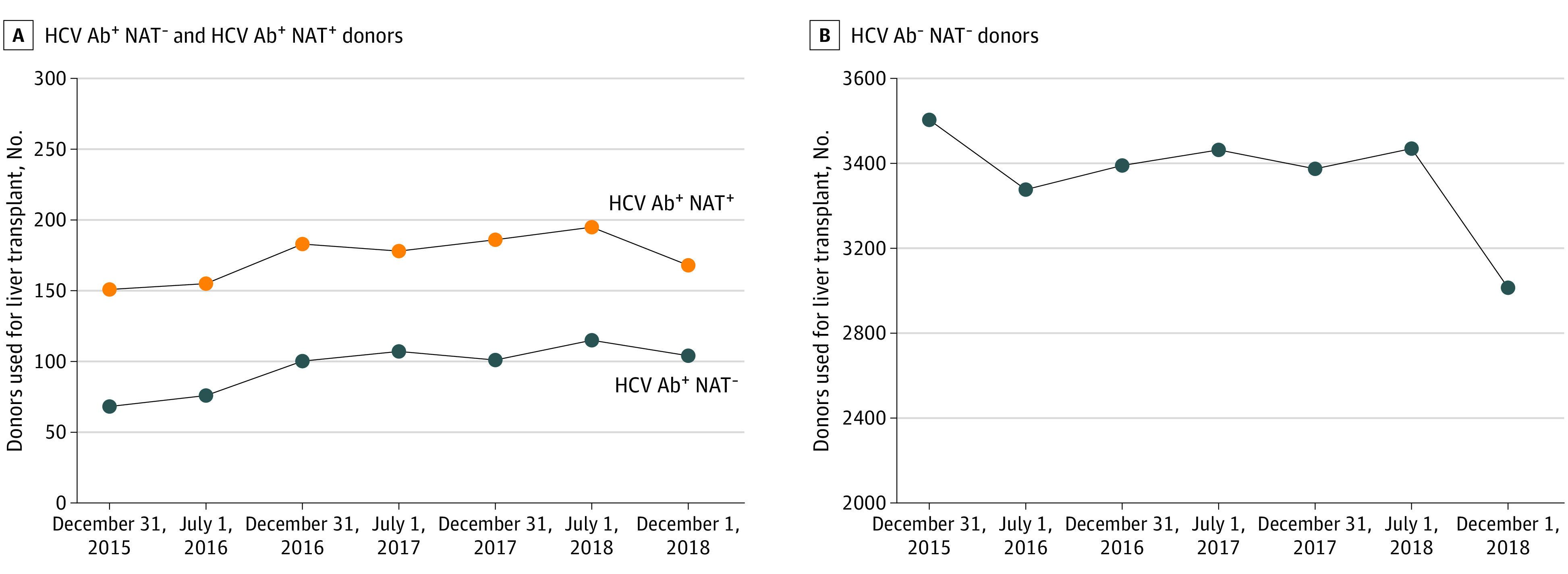
The Trend of the Number of HCV Ab^+^ NAT^−^ and HCV Ab^+^ NAT^+^ Donors and the Trend of the Number of HCV Ab^−^ NAT^−^ Donors A, This panel shows the trend of the volume of HCV Ab^+^ NAT^−^ and HCV Ab^+^ NAT^+^ donors utilized in the preceding 6 months from June 1, 2015 to December 1, 2018. Data from December 2018 was unavailable and not included. B, This panel shows the trend of the volume of HCV Ab^−^ NAT^−^ donors utilized in the preceding 6 months from June 1, 2015 to December 1, 2018. Data from December 2018 was unavailable and not included. Superscript plus sign indicates positive for HCV; superscript minus sign, negative for HCV; Ab, antibody; HCV, hepatitis C virus; NAT, nucleic acid amplification testing.

From June 1, 2015, to December 1, 2018, a total of 24 500 donors were used for LT, of which 1887 (7.7%) donors were HCV Ab positive and 22 613 (93.2%) were HCV Ab negative. During the same time period, 1280 (5.2%) donors were identified as HCV NAT positive compared with 23 220 (94.8%) HCV NAT negative. The trend of the number of HCV Ab–positive/NAT–negative, HCV Ab–positive/NAT–positive, and HCV Ab–negative/NAT–negative donors utilized in LT are shown in Figure 1.

### Clinical Characteristics: HCV Ab–Positive vs HCV Ab–Negative Donors

Compared with HCV Ab–negative donors, HCV Ab–positive donors were younger (median [IQR] age, 35 [29-46] years vs 40 [27-54] years; *P* < .001), had fewer comorbidities, such as diabetes (8.3% vs 12.0%; *P* < .001) and hypertension (25.9% vs 35.2%; *P* < .001), and were more likely to be White (81.0% vs 63.2%; *P* < .001) (Table 1). HCV Ab–positive donors were also more likely to have a history of heavy alcohol and tobacco use, cocaine use, and other drug abuse. The primary mechanism of death in HCV Ab–positive donors was drug overdose in 51.7% compared with 11.1% of the deaths (*P* < .001) in HCV Ab–negative donors.

**Table 1.** Baseline Characteristics of Donors by HCV Ab Status, June 2015 to December 2018

Characteristic	No. (%)		*P* value
	HCV Ab positive (n = 1887)	HCV Ab negative (n = 22 613)	
Age, median (IQR), y	35 (29-46)	40 (27-54)	<.001
Gender			.30
Male	1118 (59.3)	13 674 (60.5)	
Female	769 (40.8)	8939 (39.5)	
Race			<.001
White	1528 (81.0)	14 295 (63.2)	
Hispanic	139 (7.4)	3111 (13.8)	
Black or African American	202 (10.7)	4358 (19.3)	
Asian	7 (0.4)	586 (2.6)	
Other	11 (0.6)	263 (1.2)	
ABO type			<.001
A	698 (37.0)	8451 (37.4)	
AB	32 (1.7)	688 (3.0)	
B	172 (9.1)	2647 (11.7)	
O	985 (52.2)	10 827 (47.9)	
BMI, median (IQR), kg/m^2^	25.9 (23.1-29.8)	27.0 (23.5-31.5)	<.001
Hypertension	488 (25.9)	7953 (35.2)	<.001
Diabetes	156 (8.3)	2717 (12.0)	<.001
Cigarette use (>20 pack years)	298 (31.7)	4391 (19.4)	<.001
Heavy alcohol use (>2 drinks/d)	375 (19.9)	3588 (15.9)	<.001
History of cocaine use	969 (52.1)	4381 (19.6)	<.001
History of other drug abuse	1544 (83.0)	9863 (44.2)	<.001
Increased risk donor	1496 (79.3)	5340 (23.6)	<.001
Hepatitis B			
Surface antigen (+)	2 (0.1)	19 (0.1)	.75
Detectable by PCR	17 (0.9)	36 (0.2)	<.001
Core Ab (+)	340 (18.0)	902 (4.0)	<.001
HIV			
Ab (+)	1 (0.1)	27 (0.1)	.41
Detectable by PCR	1 (0.1)	16 (0.1)	.78
Liver biopsy performed	1273 (67.7)^a^	8121 (36.2)^a^	<.001
Micro-vesicular steatosis (≥5%)	629 (52.9)^b^	4346 (58.3)^b^	.001
Macro-vesicular steatosis			<.001
0%-4%	577 (46.9)^b^	2897 (37.0)^b^	
5%-30%	618 (50.2)^b^	4483 (57.2)^b^	
31%-60%	30 (2.4)^b^	400 (5.1)^b^	
>60%	5 (0.4)^b^	59 (0.8)^b^	
Mechanism of death			<.001
Drug overdose	976 (51.7)	2503 (11.1)	
Intracranial hemorrhage/stroke	295 (15.6)	6754 (29.9)	
Cardiovascular	225 (11.9)	3861 (17.1)	
Other	391 (20.7)	9495 (42.0)	
DCD	127 (7.1)	1406 (6.8)	.63
Cold ischemia time, median (IQR), h	5.9 (4.8-7.3)	5.8 (4.5-7.2)	.02
Liver DRI, median (IQR)	1.2 (1.1-1.5)	1.4 (1.2-1.7)	<.001

Values expressed as median (IQR) or No. (%) unless otherwise stated.

Abbreviations: Ab, antibody; BMI, body mass index; DCD, deceased after cardiac death; DRI, donor risk index; HCV, hepatitis C virus; IQR, interquartile range; PCR, polymerase chain reaction.

^a^ <5% missing data.

^b^ 5%-10% missing data.

### Donor Allograft Characteristics: HCV Ab–Positive vs HCV Ab–Negative Donors

A total of 67.7% of HCV Ab–positive donors underwent liver biopsy compared with 36.2% of HCV Ab–negative donors (*P* < .001) (Table 1). On biopsy, HCV Ab–positive allografts were less likely to have microvesicular (52.9% vs 58.3%; *P* = .001) and macrovesicular steatosis (53.0% vs 63.1%; *P* < .001). HCV Ab–positive allografts also had a lower median (IQR) liver donor risk index compared with HCV Ab–negative allografts (1.2 [1.1-1.5] vs 1.4 [1.2-1.7]; *P* < .001). Data on liver inflammation or liver fibrosis was not available. However, HCV Ab–positive grafts offered for LT were more likely to not be transplanted due to biopsy findings compared with HCV Ab–negative grafts 3.9% vs 2.6%.

### Comparison of HCV NAT–Positive and HCV Ab–Positive Donors

Among HCV Ab–positive donors, 1216 (64.4%) were HCV NAT positive (eTable 1 in the Supplement). A total of 671 (35.6%) of HCV Ab–positive donors were HCV NAT negative, representing cleared or treated HCV infection. Among the HCV Ab–negative donors, 22 549 (99.7%) were HCV NAT negative. Only 64 (0.3%) HCV Ab–negative donors were HCV NAT positive, which likely represented acute HCV infection.

Baseline donor and allograft characteristics of HCV NAT–positive vs HCV NAT–negative donors were similar to differences between HCV Ab–positive vs HCV Ab–negative donors and liver allografts (eTable 2 in the Supplement), and HCV Ab–positive/NAT–positive (HCV exposed + viremic) vs HCV Ab–positive/NAT–negative (HCV exposed + nonviremic) donor comparisons are shown in Table 2. Interestingly, HCV Ab–positive/NAT–positive donors were younger (34 vs 38 years old; *P* < .001) and more likely to be male (64.5% vs 49.8%; *P* < .001) compared with HCV Ab–positive/NAT–negative donors. Also, HCV Ab–positive/NAT–positive donors had fewer comorbidities (ie, diabetes and hypertension) compared with HCV Ab–positive/NAT–negative donors. On liver biopsy, there was no difference in microvesicular and macrovesicular steatosis in HCV Ab–positive/NAT–positive liver allografts compared with HCV Ab–positive/NAT–negative liver allografts. Liver donor risk index was lower in HCV Ab–positive/NAT–positive compared with HCV Ab–positive/NAT–negative grafts.

**Table 2.** Baseline Characteristics of HCV Ab–Positive Donors by HCV NAT Status, June 2015 to December 2018

Characteristic	No. (%)		*P* value
	HCV Ab positive, HCV NAT positive (n = 1216)	HCV Ab positive, HCV NAT negative (n = 671)	
Age, median (IQR), y	34 (28-43)	38 (30-52)	<.001
Gender			<.001
Male	784 (64.5)	334 (49.8)	
Female	432 (35.5)	337 (50.2)	
Race			.71
White	986 (81.1)	542 (80.8)	
Hispanic	85 (7.0)	54 (8.1)	
Black or African American	132 (10.9)	70 (10.4)	
Asian	6 (0.5)	1 (0.2)	
Other	7 (0.6)	4 (0.6)	
ABO type			.92
A	454 (37.3)	244 (36.4)	
AB	19 (1.6)	13 (1.9)	
B	110 (9.1)	62 (9.2)	
O	633 (52.1)	352 (52.5)	
BMI, median (IQR), kg/m^2^	25.6 (22.9-28.8)	27.1 (23.7-31.1)	<.001
Hypertension	256 (21.1)	232 (34.6)	<.001
Diabetes	79 (6.5)	77 (11.5)	<.001
Cigarette use (>20 pack years)	344 (28.3)	254 (37.9)	<.001
Heavy alcohol use (>2 drinks/d)	226 (18.6)	149 (22.2)	.06
History of cocaine use	630 (52.4)	339 (51.4)	.69
History of other drug abuse	1017 (84.6)	527 (80.0)	.01
Increased risk donor	1014 (83.4)	482 (71.8)	<.001
Hepatitis B			
Surface antigen (+)	1 (0.1)	1 (0.1)	.67
Detectable by PCR	9 (0.7)	8 (1.2)	.32
Core Ab (+)	177 (14.6)	163 (24.3)	<.001
HIV			
Ab (+)	1 (0.1)	0 (0.0)	.46
Detectable by PCR	1 (0.1)	0 (0.0)	.46
Liver biopsy performed	843 (69.4)^a^	430 (64.6)^a^	.03
Micro-vesicular steatosis (≥5%)	410 (51.5)^b^	219 (55.7)^b^	.17
Macro-vesicular steatosis			.52
0%-4%	391 (48.2)^a^	196 (44.0)^a^	
5%-30%	396 (48.8)^a^	222 (53.0)^a^	
31%-60%	20 (2.5)^a^	10 (2.4)^a^	
>60%	4 (0.5)^a^	1 (0.2)^a^	
Mechanism of death			<.001
Drug overdose	652 (53.6)	324 (48.3)	
Intracranial hemorrhage/stroke	168 (13.8)	127 (18.9)	
Cardiovascular	124 (10.2)	101 (15.1)	
Other	272 (22.4)	119 (17.7)	
DCD	84 (7.3)	43 (6.7)	.69
Cold ischemia time, median (IQR), h	5.9 (4.8-7.2)	5.8 (4.7-7.3)	.61
Liver DRI, median (IQR)	1.2 (1.1-1.4)	1.3 (1.2-1.6)	<.001

Values expressed as median (IQR) or No. (%) unless otherwise stated.

Abbreviations: Ab, antibody; BMI, body mass index; DCD, deceased after cardiac death; DRI, donor risk index; HCV, hepatitis C virus; IQR, interquartile range; NAT, nucleic acid amplification testing; PCR, polymerase chain reaction.

^a^ <5% missing data.

^b^ 5%-10% missing data.

### Geographic Distribution of HCV-Positive Donors Across OPTN Regions

**Figure 2.**
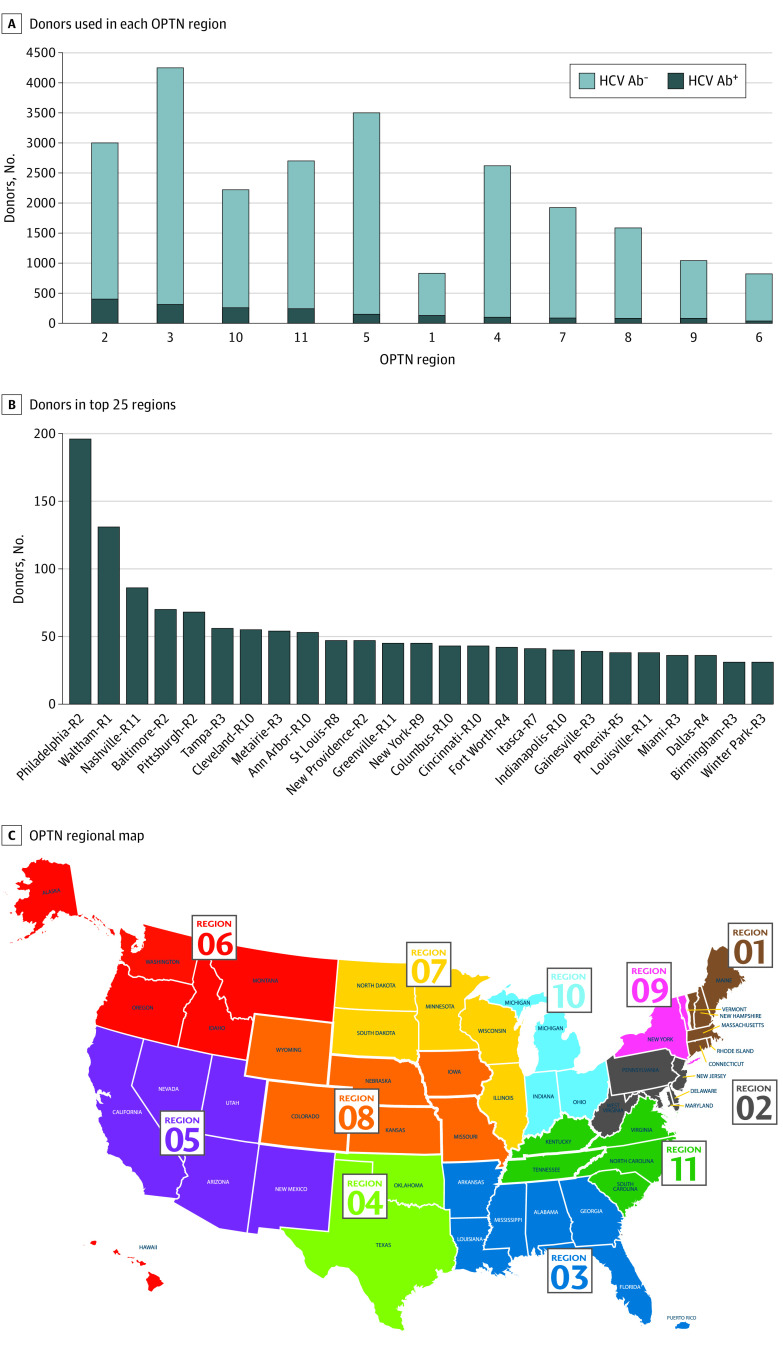
The Volume of HCV Ab^+^ Donors Utilized in Each OPTN Region and the Volume of HCV Ab^+^ Donors Utilized Sorted Based on the Top 25 OPO Centers Sorted based on the volume of HCV Ab^+^ donors used in each OPTN region. Image obtained from https://unos.org/community/regions/. Reprint with permission from UNOS. Superscript plus sign indicates positive for HCV; HCV, hepatitis C virus; OPO, organ procurement organization; OPTN, Organ Procurement and Transplantation Network.

The distribution of the number of HCV Ab–positive donors utilized in adult deceased donor LT across the 11 OPTN regions is shown in Figure 2A and eTable 3 in the Supplement. The top 4 regions in terms of total HCV Ab–positive donors (2, 3, 10, and 11) utilized 64.4% of all HCV Ab–positive donors in the United States. Region 2 accounted for the highest total utilization of HCV Ab–positive donors (21.3% of US total), and region 6 had the lowest total utilization of HCV Ab–positive donors (2.0% of US total). Figure 2C depicts the states relevant to their OPTN region.

Region 1 had the highest rate of utilization of HCV Ab–positive donors relative to their total number of LT (131 donors, 15.8% of region total) followed by region 2 (402 donors, 13.4% of region total) and region 10 (258 donors, 11.6% of region total). Region 4 had the lowest rate of utilization of HCV Ab–positive donors relative to their total number of LT (100 donors, 3.8% of region total).

eFigure 1 in the Supplement shows the distribution of the ultimate destination (recipient OPTN region) of HCV Ab–positive donor grafts from each donor OPTN region. Interestingly, certain regions (1, 2, 3, 5, 9, and 10) utilized greater than 80% of the HCV-positive donors for the same region, while other regions (4, 6, 7, and 8) utilized less than 70% of the HCV-positive donors from that particular region. In regions that utilized lower rates of HCV-positive allografts for their region, the organs usually were being utilized in nearby regions. A similar regional distribution was seen with the number of HCV NAT–positive donors utilized in LT (eFigure 2 and eTable 4 in the Supplement).

### The Volume of HCV-Positive Donors Across Organ Procurement Organization Centers

The distribution of the total number of HCV Ab–positive donors utilized in LT across the top 25 organ procurement organization (OPO) centers is shown in Figure 2B. Philadelphia, PA (196 donors), Waltham, MA (131 donors), and Nashville, TN (86 donors) were the top 3 OPO centers with respect to utilization of HCV Ab–positive donors. On the contrary, 13 OPO centers utilized less than 10 HCV Ab–positive liver allografts (eTable 5 in the Supplement). Similar findings were seen among HCV NAT–positive donors. Additionally, conversion rates of HCV Ab (positive and negative) donors from the potential to actually utilized liver donors are depicted in Table 3 (OPTN regions) and eTable 6 in the Supplement (top 25 OPO centers). Conversion rates of HCV Ab–positive/NAT–positive, Ab–positive/NAT–negative, and Ab–negative/NAT–negative donors by OPTN region are shown in eTable 7 in the Supplement. The number of HCV Ab–positive liver donors utilized by each OPTN region and OPO center was not necessarily associated with higher conversion rates at that center. However, HCV Ab–positive/NAT–positive and HCV Ab–positive/NAT–negative grafts were less likely to undergo LT locally when offered compared with HCV Ab–negative/NAT–negative grafts (approximately 46% vs 67%) (eTable 8 in the Supplement). The average countrywide conversion rates of HCV Ab (positive and negative) among the 11 OPTN regions and the top 25 OPO centers are similar.

**Table 3.** Conversion Rates of HCV Ab (+) and (-) Donors by OPTN Region

OPTN region	Total potential HCV Ab–positive donors	HCV Ab–positive donors who underwent LT	Actual conversion rate, %	Total potential HCV Ab–negative donors offered	HCV Ab–negative donors who underwent LT	Actual conversion rate, %
1	183	131	71.6	1038	699	67.3
2	530	402	75.8	3680	2601	70.7
3	377	313	83.0	4739	3935	83.0
4	155	100	64.5	3343	2520	75.4
5	238	150	63.0	4673	3355	71.8
6	56	38	67.9	1231	783	63.6
7	111	87	78.4	2541	1837	72.3
8	116	83	71.6	2157	1503	69.7
9	119	83	69.7	1377	959	69.6
10	314	258	82.2	2572	1963	76.3
11	312	242	77.6	3184	2458	77.2
OPTN region total	2511	1887	73.2^a^	30 535	22 613	72.4^a^

Values expressed as No. or %.

Abbreviations: Ab, antibody; HCV, hepatitis C virus; LT, liver transplant; OPTN, Organ Procurement and Transplantation Network.

^a^ Average conversion rate across OPTN regions.

### Deaths From Drug Overdose Across OPTN Regions

The distribution of drug overdose and deaths in HCV Ab–positive and HCV Ab–negative is shown in eTable 9 in the Supplement. For all donors, in regions 1, 2, 9, and 10, drug overdose was the cause of death for more than 20% of all donors: region 1 at 25.7%, followed by region 2 at 24.7%, region 9 at 22.2%, and region 10 at 20.9%. On the contrary, drug overdose was the least common cause of donor death in region 4 at 4.8%. Meanwhile, in regions 1, 2, 7, 9, and 10, drug overdose was the cause of death in more than 60% of all HCV Ab–positive donors (eTable 9 in the Supplement). Similar findings were seen for NAT–positive vs NAT–negative donors.

## Discussion

In this study of the SRTR database, we performed an in-depth analysis of the clinical and liver allograft characteristics and regional differences in the use of HCV Ab–positive and HCV NAT–positive donors compared with HCV-negative donors. Our data suggest that regardless of how HCV is classified (HCV Ab–positive or HCV NAT–positive), allografts from HCV-positive donors made for higher quality grafts compared with those from HCV-negative donors. HCV-positive donors were more likely to be White, which is consistent with racial/ethnic demographic characteristics of the opioid epidemic.^20^ HCV-positive donors also tended to be younger, have fewer comorbidities such as obesity, diabetes, and hypertension, less steatosis in their liver, and lower liver donor risk index. These liver allograft qualities have been associated with lower rates of biliary complications and improved rejection rates, graft survival, and overall survival.^16,17,18^ Despite this, recent studies have shown that the discard rate of HCV-positive organs remains nearly double the rate of HCV-negative organs.^19^ Donor characteristics such as age and presence of comorbidities are even more pronounced in HCV Ab–positive/NAT–positive donors compared with HCV Ab–positive/NAT–negative donors. This distinct cohort of younger HCV-positive donors are likely more optimal donors with less allograft fibrosis.^13^

We found that significant geographical variations exist in the utilization of HCV-positive donors. Four OPTN regions utilized 64.4% of all HCV Ab–positive liver allografts in the United States, with region 2 accounting for 21.3% of the HCV Ab–positive liver allografts used in the entire country. One explanation for the HCV-positive donor discrepancy is the geographical prevalence of the ongoing opioid epidemic.^20^ Organs from donors who have died of drug overdose compared with other causes of death are much more likely to be positive for HCV.^21^ Region 2 is among the highest rate of donor deaths from drug overdose, which explains the large number of HCV-positive donors that was used in this region. Our data additionally revealed somewhat unexpected variations in the utilization of HCV-positive organs. While high utilization might be expected in OPTN regions with a high median MELD score at the time of LT to maximize the pool of potential donors, the use of HCV-positive organs may not appreciably increase the organ pool in regions that have not experienced severe effects from the opioid epidemic.^22^ For example, while region 5 has the highest median MELD at transplant in the country, it only ranked fifth out of 11 regions with respect to the number of HCV-positive donors utilized.^1^ The conversion rate of HCV Ab–positive potential donors to actual donors used in LT was only 63.0% in this region, which is below the national average of 73.2%. Los Angeles, a major OPO center in region 5, only utilized 23 HCV Ab–positive donors in the time frame of the study (31st most in the country).^1^ This may also be related in part to a relative paucity of HCV-positive donors due to a lower rate of opioid overdose deaths in region 5 compared to regions such as 1, 2, 9, and 10.

The geographical distribution of the opioid epidemic does not, however, fully explain all of the utilization trends that we found in this study. For example, regions 4, 6, 7, and 8 include areas of the country that have been hit heavily by the opioid epidemic but performed the fewest transplants with HCV-positive donors.^5^ Explanations for this discrepancy are unclear but may include differences in rates of donor registration among potential donors and variations in the practices of transplant centers.^23^ A higher relative rate of non–opioid related deaths due to trauma compared with opioid overdose may occur in some regions due to variations in public safety laws, reducing the relative number of donors who die with HCV infection.^24^ That said, a higher rate of opioid-related death did not guarantee higher utilization, as we found that region 7 had among the highest rates of donor deaths from drug overdose (5th of 11) but only 4.7% of the total number of LT performed in this region was with HCV-positive donors. Region 7 also has among the highest median MELD at the time of LT, suggesting that organ demand is not the reason for the low utilization of HCV-positive donors.^25^ Finally, when we investigated the destination of the HCV-positive donor allografts in regions 4, 6, 7, and 8, we found that more than 30% of the HCV-positive donor allografts in these regions were being used in nearby regions, suggesting that some of these organs were being passed up locally.

Lack of utilization of HCV-positive organs in certain high MELD regions may also be explained by higher barriers to HCV treatment in different areas of the country, which can be related to state Medicaid policies and the socioeconomic level of the region.^26^ While there are currently fewer barriers to treatment compared with the early DAA era, in some states, insurers—especially Medicaid programs—have strict requirements for approval of DAA therapy, including the need for specialist prescribers, required periods of abstinence from drugs and alcohol, and a restriction of treatment to patients with higher stages of fibrosis.^27^ The more stringent the Medicaid criteria for HCV treatment, the less likely it is for HCV treatment to be initiated.^28^ The converse is true in states like Massachusetts and New Hampshire (both located in region 1), where there is a lower requirement to HCV treatment and initiation of treatment is easier.^13^ The socioeconomic status of the region also matters as areas with patients of lower socioeconomic status are more likely to have Medicaid insurance coverage, which has a much higher treatment denial rate compared to commercial insurance.^29^ Removing financial/insurance-related barriers is key in promoting the use of HCV-positive donors as it establishes a stable setup for post-LT HCV treatment, which provides assurance to transplant centers and patients that the posttransplant course will not be complicated by effects of untreated posttransplant HCV.^28^

A final explanation for the regional variation in HCV-positive donor utilization may be variations in patient perception of transplantation with organs from an HCV-positive donor. A patient survey conducted by Couri et al^30^ in patients undergoing kidney transplant found that 90% were aware of HCV, but only 60% knew that it was curable, and only 46% were willing to receive an HCV-positive organ. As such, certain centers may be better at providing patients with education about the benefits of accepting HCV-positive donors. As physicians, we must be diligent in guiding our patients by highlighting the risks and benefits that come with accepting an HCV-positive donor and providing adequate informed consent.^21^ One of the potential avenues to accomplish this task is by using social media as a way to disseminate knowledge about the use of HCV-positive donors to physicians and patients across the country, remove the social stigma around the use of HCV-positive grafts, and improve perception.^31,32^

### Strengths and Limitations

The strengths of this study are that we were able to depict the regional utilization of HCV-positive donors in liver transplantation on a fairly granular level using a large national database. As such, we could highlight and document areas that may be underusing HCV-positive grafts, which we hope may enable future advancements to bridge the gap. This study has several limitations. We did not report outcome data of HCV-positive grafts as such studies would need to also focus on recipient characteristics, and our study was primarily descriptive aimed at highlighting donor characteristics and utilization. Additionally, although we can speculate on the reasons behind the differences in regional utilization through correlation within our data (ie, incidences of drug overdose), we suspect that there are multiple confounders that are unaccounted for in the SRTR database. These may include differing institutional, physician, and patient’s psychosocial and efficacy perceptions regarding HCV-positive grafts among different regions.

## Conclusions

In summary, as a result of the opioid epidemic and a related increase in the number of drug overdose deaths, the utilization of HCV-positive donors is a useful approach to expand the donor pool. In this study, we found that HCV-positive donors tend to be healthier and donate superior liver allografts compared with HCV-negative donors. This association was even more pronounced in HCV viremic donors. Despite this finding, there exist substantial variations in the utilization of these donors across the country that are not entirely explained by the geography of the opioid epidemic. Lowering the barriers to HCV treatment by improving insurance coverage, eliminating outdated requirements that delay timely initiation of treatment, and improving public perception of the use of these organs through education and media may be the ideal way to improve the utilization of these organs.

## Notes

Supplement. **eTable 1. **Donor HCV Ab and NAT status **eTable 2. **Baseline characteristics of donors by HCV NAT status (mid 2015-2018) **eTable 3. **The volume of HCV Ab+ donors utilized in each OPTN region **eTable 4. **The volume of HCV NAT+ donors utilized in each OPTN region **eTable 5. **The volume of HCV Ab+ donors utilized in each OPO center **eTable 6. **Conversion rates of HCV Ab (+) and (-) donors by the top 25 OPO centers **eTable 7. **Conversion rates of HCV Ab+/NAT+, Ab+/NAT-, and HCV Ab- donors by OPTN region **eTable 8. **Ultimate destination of HCV Ab+/NAT+, HCV Ab+/NAT-, and HCV Ab-/NAT- grafts **eTable 9. **Distribution of Drug Overdose by HCV Ab status **eFigure 1. **Distribution of the ultimate destination (recipient OPTN region) of HCV Ab (+) donor grafts from each donor OPTN region **eFigure 2. **The volume of HCV NAT (+) donors utilized in each OPTN region **eMethods. **Data Collection **eReferences. **
