# Supplementary material for: Donor Characteristics and Regional Differences in the Utilization of HCV-Positive Donors in Liver Transplantation
Source: JAMA Netw Open. 2020 Dec 4;3(12):e2027551. doi: 10.1001/jamanetworkopen.2020.27551 (PMC7718602; doi:10.1001/jamanetworkopen.2020.27551)
Supplement: Supplement. — eTable 1. Donor HCV Ab and NAT status eTable 2. Baseline characteristics of donors by HCV NAT status (mid 2015-2018) eTable 3. The volume of HCV Ab+ donors utilized in each OPTN region eTable 4. The volume of HCV NAT+ donors utilized in each OPTN region eTable 5. The volume of HCV Ab+ donors utilized in each OPO center eTable 6. Conversion rates of HCV Ab (+) and (-) donors by the top 25 OPO centers eTable 7. Conversion rates of HCV Ab+/NAT+, Ab+/NAT-, and HCV Ab- donors by OPTN region eTable 8. Ultimate destination of HCV Ab+/NAT+, HCV Ab+/NAT-, and HCV Ab-/NAT- grafts eTable 9. Distribution of Drug Overdose by HCV Ab status eFigure 1. Distribution of the ultimate destination (recipient OPTN region) of HCV Ab (+) donor grafts from each donor OPTN region eFigure 2. The volume of HCV NAT (+) donors utilized in each OPTN region eMethods. Data Collection eReferences. [file jamanetwopen-e2027551-s001.pdf]

Supplemental Online Content

Da BL, Ezaz G, Kushner T, et al. Donor characteristics and regional differences in the utilization of HCV-positive donors in liver transplantation. *JAMA Netw Open*. 2020;3(12):e2027551. doi:10.1001/jamanetworkopen.2020.27551

eTable 1. Donor HCV Ab and NAT status

eTable 2. Baseline characteristics of donors by HCV NAT status (mid 2015-2018)

eTable 3. The volume of HCV Ab+ donors utilized in each OPTN region

eTable 4. The volume of HCV NAT+ donors utilized in each OPTN region

eTable 5. The volume of HCV Ab+ donors utilized in each OPO center

eTable 6. Conversion rates of HCV Ab (+) and (-) donors by the top 25 OPO centers

eTable 7. Conversion rates of HCV Ab+/NAT+, Ab+/NAT-, and HCV Ab- donors by OPTN region

eTable 8. Ultimate destination of HCV Ab+/NAT+, HCV Ab+/NAT-, and HCV Ab-/NAT- grafts

eTable 9. Distribution of Drug Overdose by HCV Ab status

eFigure 1. Distribution of the ultimate destination (recipient OPTN region) of HCV Ab (+) donor grafts from each donor OPTN region

eFigure 2. The volume of HCV NAT (+) donors utilized in each OPTN region

eMethods. Data Collection

eReferences.

This supplemental material has been provided by the authors to give readers additional information about their work.

© 2020 Da BL et al. *JAMA Network Open.*

eTable 1. Donor HCV Ab and NAT status

	HCV Ab (+)<br>(n = 1,887)	HCV Ab (-)<br>(n = 22,613)
HCV NAT (+)<br>(n = 1,280)	1,216<br>(64.4%)	64<br>(0.3%)
HCV NAT (-)<br>(n = 23,220)	671<br>(35.6%)	22,549<br>(99.7%)

June 1st, 2015 to December 1st, 2018
Abbreviations: HCV, hepatitis C virus; Ab, antibody; NAT, nucleic acid amplification

© 2020 Da BL et al. *JAMA Network Open.*

eTable 2. Baseline characteristics of donors by HCV NAT status (mid 2015-2018)

	HCV NAT (+)<br>(n = 1,280)	HCV NAT (-)<br>(n = 23,220)	*P*
Age, years	34 (28-42)	40 (27-54)	< 0.001
Gender			0.009
Male	817 (63.8%)	13,975 (60.2%)	
Female	462 (36.2%)	9,245 (39.8%)	
Race			< 0.001
White	1,042 (81.4%)	14,781 (63.7%)	
Hispanic	88 (6.9%)	3,162 (13.6%)	
Black or African American	137 (10.7%)	4,423 (19.1%)	
Asian	6 (0.5%)	587 (2.5%)	
Other	7 (0.6%)	267 (1.2%)	
ABO type			< 0.001
A	487 (38.1%)	8,662 (37.3%)	
AB	20 (1.6%)	700 (3.0%)	
B	114 (8.9%)	2,705 (11.7%)	
O	659 (51.5%)	11,153 (48.0%)	
BMI	27.0 (23.5-31.5)	25.6 (22.9-28.8)	< 0.001
Hypertension	263 (20.5%)	8,179 (35.2%)	< 0.001
Diabetes	82 (6.4%)	2,791 (12.0%)	< 0.001
Cigarette use (>20 pack years)	354 (27.7%)	4,635 (20.0%)	< 0.001
Heavy alcohol use (>2 drinks/day)	235 (18.4%)	3,728 (16.1%)	0.03
History of cocaine use	667 (52.7%)	4,683 (20.4%)	< 0.001
History of other drug abuse	1,067 (84.4%)	10,340 (45.1%)	< 0.001
Increased risk donor	1,069 (83.5%)	5,767 (24.8%)	< 0.001
Hepatitis B surface antigen (+)	1 (0.1%)	20 (0.1%)	0.92
Hepatitis B detectable by PCR	11 (0.9%)	42 (0.2%)	< 0.001
Hepatitis B core Ab (+)	178 (13.9%)	1,064 (4.6%)	< 0.001
HIV Ab (+)	2 (0.2%)	26 (0.1%)	0.65
HIV detectable by PCR	3 (0.2%)	14 (0.1%)	0.02
Liver biopsy performed	883/1278 (69.1%)	8,515/23,070 (36.9%)	< 0.001
Micro-vesicular steatosis present	429/834 (51.4%)	4,546/7,813 (58.2%)	< 0.001
Macro-vesicular steatosis			< 0.001
0-4%	417/851 (49.0%)	3,057/8,218 (37.2%)	
5-30%	410/851 (48.2%)	4,691/8,218 (57.1%)	
31-60%	20/851 (2.4%)	410/8,218 (5.0%)	
> 60%	4/851 (0.5%)	60/8,218 (0.7%)	
Mechanism of death			< 0.001
Drug overdose	691 (54.0%)	2,788 (12.0%)	
Intracranial hemorrhage/Stroke	173 (13.5%)	6,876 (29.6%)	

Cardiovascular<br>Other	133 (10.4%)<br>283 (22.1%)	3,953 (17.0%)<br>9,603 (41.4%)	
DCD	93 (7.6%)	1,440 (6.8%)	0.24
Cold ischemia time, hours	5.9 (4.8-7.2)	5.8 (4.5-7.2)	0.02

Values expressed as median (IQR) or n (%) unless otherwise stated.
Abbreviations: Ab, antibody; HCV, hepatitis C virus; BMI, body mass index; PCR, polymerase chain reaction; DCD, deceased after cardiac death.

© 2020 Da BL et al. *JAMA Network Open.*

eTable 3. The volume of HCV Ab (+) donors utilized in each OPTN region.

Region	2	3	10	11	5	1	4	7	8	9	6
Total HCV AB (+)	402	313	258	242	150	131	100	87	83	83	83
Total HCV AB (-)	2601	3935	1963	2458	3355	699	2520	1837	1503	959	783
Percent HCV Ab (+)	13.4	7.4	11.6	9.0	4.3	15.8	3.8	4.5	5.2	8.0	9.6

Sorted based on the volume of HCV Ab (+) donors utilized in each OPTN region.
Abbreviations: HCV, hepatitis C virus; OPTN, Organ Procurement, and Transplantation Network

© 2020 Da BL et al. *JAMA Network Open.*

eTable 4. The volume of HCV NAT (+) donors utilized in each OPTN region.

Region	2	3	10	11	5	1	4	7	8	9	6
Total HCV NAT (+)	266	210	182	176	97	92	68	63	56	48	22
Total HCV NAT (-)	2,737	4,038	2,039	2,524	3,408	738	2,552	1,523	1,868	994	799
Percent HCV NAT (+)	8.9	4.9	8.2	6.5	2.8	11.1	2.6	4.0	2.9	4.6	2.7

Sorted based on the volume of HCV NAT (+) donors utilized in each OPTN region.
Abbreviations: HCV, hepatitis C virus; OPTN, Organ Procurement, and Transplantation Network

© 2020 Da BL et al. *JAMA Network Open.*

eTable 5. The volume of HCV Ab+ donors utilized in each OPO center

Rank	City	Number	% of OPO Total
1	Philadelphia - R2	196	16.1%
2	Waltham - R1	131	15.8%
3	Nashville - R11	86	10.9%
4	Baltimore - R2	70	14.6%
5	Pittsburgh - R2	68	12.7%
6	Tampa - R3	56	9.4%
7	Cleveland - R10	55	13.9%
8	Metairie - R3	54	10.1%
9	Ann Arbor - R10	53	7.5%
10	St Louis - R8	47	10.5%
11	New Providence - R2	47	10.5%
12	Greenville - R11	45	10.3%
13	New York - R9	45	6.3%
14	Columbus - R10	43	14.6%
15	Cincinnati - R10	43	22.2%
16	Fort Worth - R4	42	4.7%
17	Itasca - R7	41	4.2%
18	Indianapolis - R10	40	8.7%
19	Gainesville - R3	39	9.8%
20	Phoenix - R5	38	6.9%
21	Louisville - R11	38	12.5%
22	Miami - R3	36	8.3%
23	Dallas - R4	36	3.6%
24	Birmingham - R3	31	6.9%
25	Winter Park - R3	31	8.4%
26	Norcross - R11	28	3.3%
27	Virginia Beach - R11	28	7.9%
28	Bellevue - R6	28	5.9%
29	San Ramon - R5	24	3.0%
30	Maumee - R10	24	14.1%
31	Los Angeles - R5	23	2.2%
32	Falls Church - R11	21	6.5%
33	San Diego - R5	20	7.0%
34	Milwaukee - R7	20	9.5%
35	Albany - R9	18	14.5%
36	Charleston - R11	18	4.5%
37	Madison - R7	18	5.4%
38	Charlotte - R11	17	6.7%
39	Las Vegas - R5	17	6.0%
40	Flowood - R3	16	7.2%
41	Westwood - R8	14	2.7%
42	Oklahoma City - R4	14	3.8%
43	Guaynabo - R3	13	4.9%
44	Rochester - R9	12	11.3%
45	Denver - R8	11	4.0%
46	Albuquerque - R5	11	8.7%
47	Cordova - R11	10	6.0%
48	Little Rock - R3	9	6.2%
49	West Sacramento - R5	9	4.6%
50	North Liberty - R8	8	6.1%
51	Minneapolis - R7	8	2.0%
52	Buffalo - R9	8	8.4%

© 2020 Da BL et al. *JAMA Network Open.*

53	Portland - R6	8	2.9%
54	San Antonio - R4	8	2.1%
55	Salt Lake City - R5	8	3.5%
56	Omaha - R8	3	1.8%
57	Honolulu - R6	2	2.7%
58	Maitland - R3	0	0.0%
59	Covington - R3	0	0.0%
60	Williamsville - R9	0	0.0%

© 2020 Da BL et al. *JAMA Network Open.*

eTable 6. Conversion rates of HCV Ab (+) and (-) donors by the top 25 OPO centers

OPO	Total potential HCV Ab+ donors	HCV Ab+ donors who underwent LT	Actual conversion rate	Total potential HCV Ab- donors	HCV Ab- donors who underwent LT	Actual conversion rate
Philadelphia	281	196	69.8%	1,574	1,021	64.9%
Waltham	183	131	71.6%	1,038	699	67.3%
Nashville	98	86	87.8%	860	701	81.5%
Baltimore	82	70	85.4%	474	411	86.7%
Pittsburgh	86	68	79.1%	657	466	70.9%
Tampa	65	56	86.2%	681	537	78.9%
Cleveland	69	55	79.7%	447	340	76.1%
Metairie	60	54	90.0%	521	483	92.7%
Ann Arbor	74	53	71.6%	966	654	67.7%
St Louis	54	47	87.0%	592	450	76.0%
New Providence	56	47	83.9%	551	399	72.4%
Greenville	62	45	72.6%	566	391	69.1%
New York	60	45	75.0%	883	672	76.1%
Columbus	49	43	87.8%	316	252	79.7%
Cincinnati	44	43	97.7%	166	151	91.0%
Fort Worth	56	42	75.0%	1,143	844	73.8%
Itasca	56	41	73.2%	1,283	933	72.7%
Indianapolis	49	40	81.6%	491	420	85.5%
Gainesville	47	39	83.0%	442	358	81.0%
Phoenix	61	38	62.3%	711	511	71.9%
Louisville	44	38	86.4%	332	266	80.1%
Miami	48	36	75.0%	497	398	80.1%
Dallas	50	36	72.0%	1,171	952	81.3%
Birmingham	39	31	79.5%	480	417	86.9%
Winter Park	41	31	75.6%	488	337	69.1%
Top 25 total	1,814	1,411	79.6%*	17,330	13,063	77.3%*

Values expressed as n or %; * = average conversion rate across top 25 OPO
Abbreviations: HCV, hepatitis C virus; Ab, antibody; NAT, nucleic acid amplification

eTable 7. Conversion rates of HCV Ab+/NAT+, Ab+/NAT-, and HCV Ab- donors by OPTN region

OPTN region	Total potential HCV Ab-/NAT- donors	HCV Ab-/NAT- donors who underwent LT	Actual conversion rate (%)	Total potential HCV Ab+/NAT+ donors	HCV Ab+/NAT+ donors who underwent LT	Actual conversion rate (%)	Total potential HCV Ab+/NAT- donors	HCV Ab+/NAT- donors who underwent LT	Actual conversion rate (%)
1	1,033	694	67.2%	121	87	71.9%	62	44	71.0
2	3,661	2,587	70.7%	334	252	75.4%	196	150	76.5
3	4,727	3,927	83.1%	249	202	81.1%	128	111	86.7
4	3,340	2,518	75.4%	96	66	68.8%	59	34	57.6
5	4,667	3,350	71.8%	154	92	59.7%	84	58	69.0
6	1,230	782	63.6%	34	21	61.8%	22	17	77.3
7	2,537	1,833	72.3%	65	52	80.0%	46	35	76.1
8	2,150	1,499	69.7%	82	59	72.0%	34	24	70.6
9	1,374	956	69.6%	67	45	67.2%	52	38	73.1
10	2,564	1,957	76.3%	209	176	84.2%	105	82	78.1
11	3,170	2,446	77.2%	215	164	76.3%	96	78	81.3
OPTN region total	30,453	22,549	72.5%*	1,626	1,216	72.6%*	884	671	74.3%*

Values expressed as n or %; * = average conversion rate across OPTN regions
Abbreviations: HCV, hepatitis C virus; Ab, antibody; NAT, nucleic acid amplification

© 2020 Da BL et al. *JAMA Network Open.*

eTable 8. Ultimate destination of HCV Ab+/NAT+, HCV Ab+/NAT-, and HCV Ab-/NAT- grafts

		Underwent LT			
	Offered for LT	Locally	Regionally	Nationally	Total
HCV Ab+, NAT+	1,626	553 (45.5%)	439 (36.1%)	224 (18.4%)	1,216
HCV Ab+, NAT-	884	307 (45.8%)	250 (37.3%)	114 (17.0%)	671
HCV Ab-, NAT-	30,453	15,013 (66.6%)	6,705 (29.7%)	831 (3.7%)	22,549

Abbreviations: HCV, hepatitis C virus; Ab, antibody; NAT, nucleic acid amplification

eTable 9. Distribution of Drug Overdose by HCV Ab status

	Total Deaths in the Region	Drug OD Total [1]	Drug OD HCV Ab (+) [2]	Drug OD HCV Ab (-) [3]
**Region 1**	830	213 (25.7%)	79 (60.3%)	134 (19.2%)
**Region 2**	3003	743 (24.7%)	250 (62.2%)	493 (19.0%)
**Region 3**	4248	481 (11.3%)	155 (49.5%)	326 (8.3%)
**Region 4**	2620	126 (4.8%)	13 (13.0%)	113 (4.5%)
**Region 5**	3505	294 (8.4%)	41 (27.3%)	253 (7.5%)
**Region 6**	821	90 (11.0%)	14 (36.8%)	76 (9.7%)
**Region 7**	1924	290 (15.1%)	57 (65.5%)	233 (12.7%)
**Region 8**	1586	205 (12.9%)	42 (50.6%)	163 (10.8%)
**Region 9**	1042	231 (22.2%)	51 (61.4%)	180 (18.8%)
**Region 10**	2221	465 (20.9%)	167 (64.7%)	298 (15.2%)
**Region 11**	2700	341 (12.6%)	107 (44.2%)	234 (9.5%)

June 2015 to December 2018; Value expressed as n (%) or n as appropriate
[1] expressed as n (%) of total deaths in the specific region; [2] expressed as n (%) of HCV Ab (+) deaths in the specific region; [3] expressed as n (%) of HCV Ab (-) deaths in the specific region
Abbreviations: OD, overdose; HCV, hepatitis C virus; Ab, antibody

© 2020 Da BL et al. *JAMA Network Open.*

Donor OPTN Region

Recipient OPTN Region	1	2	3	4	5	6	7	8	9	10	11
1	**90.8**	3.0	0.6	4.0	0	0	4.6	2.4	3.6	0.8	4.1
2	0	**85.6**	1.0	0	0	0	2.3	4.8	2.4	1.9	4.1
3	0	0.5	**84.0**	8.0	1.3	0	8.0	7.2	0	0.8	2.1
4	0.8	0.2	1.3	**68.0**	0	2.6	1.1	2.4	1.2	0.4	1.7
5	1.5	0.2	2.2	8.0	**98.0**	26.3	5.7	4.8	1.2	3.9	2.1
6	0	0	0	00	0	**68.4**	0	0	0	0	0
7	0.8	0.7	1.0	3.0	0	0	**63.2**	3.6	0	1.2	1.2
8	0	0.2	0.6	2.0	0	2.6	2.3	**62.7**	0	1.2	0
9	4.6	4.7	5.4	3.0	0	0	5.7	8.4	**88.0**	3.5	7.0
10	1.5	4.2	2.6	2.0	0.7	0	6.9	2.4	3.6	**86.0**	3.3
11	0	0.5	1.3	2.0	0	0	0	1.2	0	0.4	**74.4**

eFig. 1. Distribution of the ultimate destination (recipient OPTN region) of HCV Ab (+) donor grafts from each donor OPTN region.
Abbreviations: HCV, hepatitis C virus; OPTN, Organ Procurement and Transplantation Network

© 2020 Da BL et al. *JAMA Network Open.*

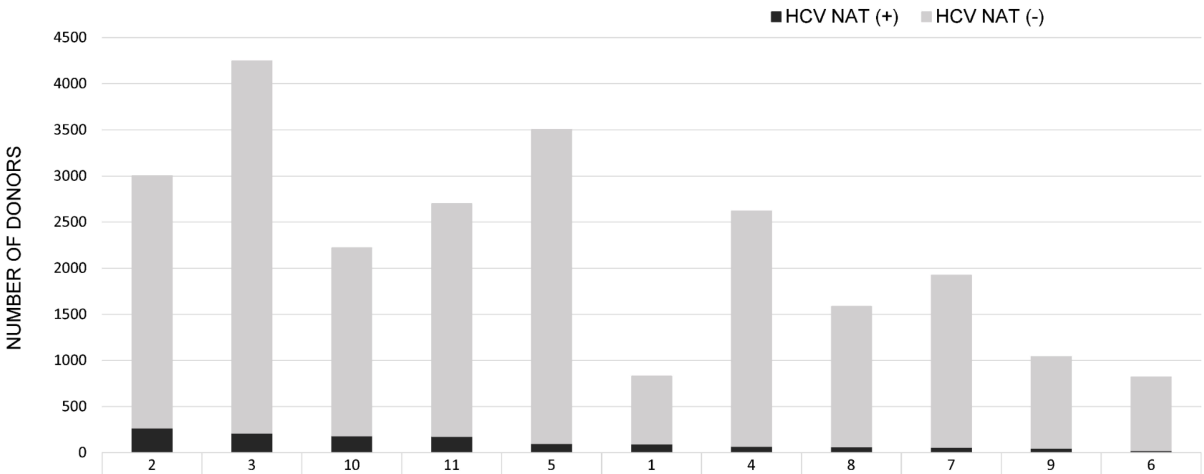

	2	3	10	11	5	1	4	8	7	9	6
HCV NAT (-)	2,737	4,038	2,039	2,524	3,408	738	2,552	1,523	1,868	994	799
HCV NAT (+)	266	210	182	176	97	92	68	63	56	48	22

OPTN REGION

eFig. 2. The volume of HCV NAT (+) donors utilized in each OPTN region.
Sorted based on the volume of HCV NAT (+) donors utilized in each OPTN region.
Abbreviations: HCV, hepatitis C virus; OPTN, Organ Procurement, and Transplantation Network

© 2020 Da BL et al. *JAMA Network Open.*

eMethods. Data Collection

Donor demographics (age, body mass index, sex, race), clinical (history of hypertension, diabetes, alcohol use, drug use, classification as a deceased after cardiac death (DCD) donor or as an increased risk donor, mechanism of death), laboratory (HIV status, hepatitis B status) characteristics were collected. Prior exposure to hepatitis B virus (HBV) was defined as testing positive for hepatitis B core Ab. Increased risk donor was defined as per the Centers for Disease Control and Prevention 2013 criteria.[1] Anyone with one of 11 possible risk factors for HIV, HBV, or HCV was considered an increased risk donor.[1] Graft characteristics such as the performance of a liver biopsy prior to transplant, presence of micro-vesicular and/or macro-vesicular steatosis, and cold ischemia time were also collected. Micro-vesicular steatosis was determined to be present if ≥5% micro-vesicular fat was present on biopsy. Macro-vesicular steatosis was graded as follows: normal (0-4%), mild (5-30%), moderate (31-60%), severe (>60%) steatosis on biopsy.[2] Conversion rate was defined as the number of donors who underwent actual LT relative to the number of total potential donors. Data regarding HCV positive donor usage was collected for each OPTN region and organ procurement organization (OPO) center.

Donor risk index (DRI) was calculated for each donor by the formula shown below.[3] A DRI ≤ 1 was considered an excellent donor with historical 3-year graft survival of greater than 80% while a DRI > 2 was considered a poor donor with 3-year graft survival of approximately 60%. Donor risk index = exp[(0.154 if 40≤age<50)+(0.274 if 50≤age<60)+(0.424 if 60≤age<70)+(0.501 if 70≤age)+(0.079 if COD=anoxia)+(0.145 if COD=CVA)+(0.184 if COD=other)+(0.176 if race=African American)+(0.126 if race=other)+(0.411 if DCD)+(0.422 if partial/split)+(0.066 ((170–height)/10))+(0.105 if regional share)+(0.244 if national share)+(0.010×cold time)

eREFERENCES

1. Seem DL, Lee I, Umscheid CA, Kuehnert MJ, United States Public Health S. PHS guideline for reducing human immunodeficiency virus, hepatitis B virus, and hepatitis C virus transmission through organ transplantation. *Public Health Rep*. Jul 2013;128(4):247-343. doi:10.1177/003335491312800403
2. Chu MJ, Dare AJ, Phillips AR, Bartlett AS. Donor Hepatic Steatosis and Outcome After Liver Transplantation: a Systematic Review. *J Gastrointest Surg*. Sep 2015;19(9):1713-24. doi:10.1007/s11605-015-2832-1
3. Feng S, Goodrich NP, Bragg-Gresham JL, et al. Characteristics associated with liver graft failure: the concept of a donor risk index. *Am J Transplant*. Apr 2006;6(4):783-90. doi:10.1111/j.1600-6143.2006.01242.x

© 2020 Da BL et al. *JAMA Network Open.*
